# Source Activity Correlation Effects on LCMV Beamformers in a Realistic Measurement Environment

**DOI:** 10.1155/2012/190513

**Published:** 2012-04-29

**Authors:** Paolo Belardinelli, Erick Ortiz, Christoph Braun

**Affiliations:** ^1^MEG Center, University of Tübingen, Otfried Mueller Street 47, 72076 Tübingen, Germany; ^2^CIMeC, Center of Mind/Brain Sciences, University of Trento, Via Delle Regole 101, 38123 Mattarello, Italy; ^3^DiSCoF, Department of Cognitive and Educational Sciences, University of Trento, Corso Bettini no. 31, 38068 Rovereto, Italy

## Abstract

In EEG and MEG studies on brain functional connectivity and source interactions can be performed at sensor or source level. Beamformers are well-established source-localization tools for MEG/EEG signals, being employed in source connectivity studies both in time and frequency domain. However, it has been demonstrated that beamformers suffer from a localization bias due to correlation between source time courses. This phenomenon has been ascertained by means of theoretical proofs and simulations. Nonetheless, the impact of correlated sources on localization outputs with real data has been disputed for a long time. In this paper, by means of a phantom, we address the correlation issue in a realistic MEG environment. Localization performances in the presence of simultaneously active sources are studied as a function of correlation degree and distance between sources. A linear constrained minimum variance (LCMV) beamformer is applied to the oscillating signals generated by the current dipoles within the phantom. Results show that high correlation affects mostly dipoles placed at small distances (1, 5 centimeters). In this case the sources merge. If the dipoles lie 3 centimeters apart, the beamformer localization detects attenuated power amplitudes and blurred sources as the correlation level raises.

## 1. Introduction

MEG and EEG provide noninvasive imaging over the whole brain with an excellent temporal resolution. The high temporal resolution qualifies these methods for the study of functional connectivity based on correlated activity. Over the last three decades, several algorithms have been developed for brain source localization [1]. Besides recent Bayesian approaches which employ iterative (and therefore computationally demanding) inversion schemes [2–4], beamformers [5, 6] still hold as one of the most reliable inversion schemes for source localization both in time and frequency domain [7–15]. Beamformers are data-dependent spatial filters. In order to pass from sensor signals to brain source activity, they employ filters which rely on the signal covariance (time domain) or the cross-spectrum density matrix (frequency domain). Moreover, beamformers assume uncorrelated source timecourses. The covariance estimation is therefore forced to be diagonal. This may induce a bias both on location and intensity of the detected sources [16]. Some findings have shown that this assumption produces no evident bias with certain data sets. On the other hand, other studies have demonstrated that it may induce relevant biases when the level of correlation between sources and the signal-to-noise Ratio are high [17, 18]. A dual-core beamformer that takes into account the correlation effects between two sources has been implemented by Brookes et al. [19]. However, this modified beamformer implies the use of *a priori *information which is not always available. The correlation effects are particularly disruptive when analyzing brain-induced or spontaneous activity in the frequency domain. In fact, some possible remedies for modeling correlation effects have been proposed for the study of evoked potential in the time domain [20, 21]. However, these approaches based on Bayesian theory call for a strong assumption about what is “signal” and what is “noise/spontaneous activity” within the time span of our data. This is clearly not possible in presence of data generated by spontaneous or induced brain activity.

In this paper, we have set true current dipoles within a phantom and measured the MEG signals generated by sources with various levels of mutual correlation located at different depths and mutual distances. The goal of the paper was to elucidate how correlation affects beamformer results in a realistic measurement environment. For this aim, we have localized the oscillating sources both in time and frequency domain by means of a linear constrained minimum variance (LCMV) beamformer. Since the localization results appeared extremely similar, we focused on the frequency domain which is a power implementation of dynamic imaging of coherent sources (DICS) [10].

## 2. Materials and Methods

### 2.1. Forward Solution

#### 2.1.1. Phantom Description

In this experiment the CTF phantom model PN900-0017 was employed. This phantom consists of a 65 mm acrylic sphere, filled with saline water at 0.8% concentration, and based on an empty vertical acrylic tube. This tool emulates the brain volume conductor and the conductive medium around it. The brain current dipoles are simulated by a twisted pair of isolated wires, with open ends, encased in a glass tube. These tubes enter the sphere from the inferior part through a grid of holes that allows the dipoles to be located at different positions on the horizontal plane.

#### 2.1.2. Head Coordinate System

To define the head coordinate system, three coils are vertically located at standard positions on the surface of the phantom sphere. The three locations loosely correspond to the fiducial points commonly used with human subjects: nasion (NAS), left periauricular point (LPA), and right periauricular point (RPA). The origin is considered the midpoint between RPA and LPA. All of the three points lie on the central horizontal plane of the sphere. The *X* axis points directly to NAS, and the *Y* axis is orthogonal to it, pointing approximately towards LPA. The *Z* axis is orthogonal to the *XY* plane, pointing at the top of the sphere.

In order to define the head localization, the three small coils (NAS, LPA, and RPA) generate a magnetic dipole signal. The center of the magnetic dipole coil is 50 mm above the plane containing the center of all three head localization coils. The dipole coil is an 11-turn single-layer air core solenoid wound on a 1.6 mm diameter mandrill. The magnetic field generated by a coil is different from the magnetic field generated by a current dipole. As a result, a different localization calculation is used for the magnetic dipole phantom.

#### 2.1.3. Dipole Locations

**Figure 1:**
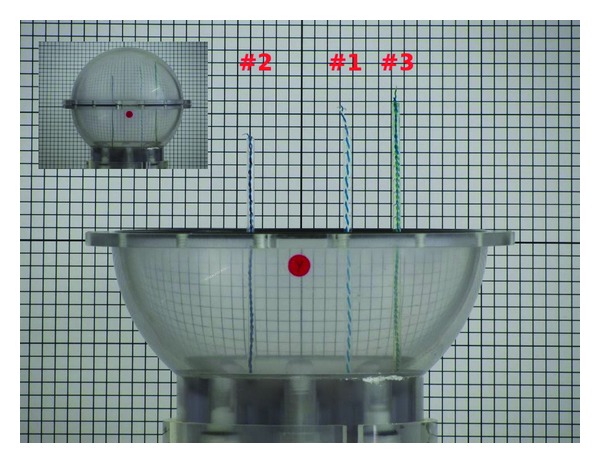
Setting of the three dipoles within the coverless phantom. Squares have a 5 mm side. In the left upper corner, the complete setup is shown.

Two different dipole configurations were considered: one with two parallel dipoles (#1 and #2) placed on the same coronal plane in the two different hemispheres (Figure 1). The dipoles had different distances from the surface (2 and 3 cm, resp.). The distance between the dipoles was 3 centimeters. In the second configuration the two dipoles (#1 and #3) were placed in the left hemisphere at a slightly height (2 and 0.5 cm from the surface, resp.), with dipole #3 more external with respect to #1. Their distance in this case was 1.5 centimeters.

#### 2.1.4. Simulated Electric Signals

The dipoles' electric signals were divided into 200 epochs (100 epochs with oscillating dipoles, 100 with inactive ones) with a length of 0.8 seconds. The time courses were generated synthetically from a frequency distribution centered at 10 Hz, controlled for the desired correlation levels, and then commuted into electric signals by means of a digital to analog converter (DAC).

A computer with a DAC board Adlink ACL-6126 was used, with one independent channel per dipole, with alternating current output in a range of ± 5 *μ*A. Since we placed one dipole (#3, Figure 1) on a location which can be roughly associated to the sensorimotor region, a typical range of electrical activity for somatosensory responses was used [22]. Our DAC sampling rate was 200 Hz, the MEG sampling rate was 293 Hz. The DAC board employed for our experiment has bipolar outputs, with a common ground. Thus, while the voltage output was bipolar, the pair was not allowed to float.

Under the conditions mentioned above, the ideal dipole current drivers are optically isolated current sources; however, in the absence of this option, the best solution was (1) to use a DAC with differential outputs (range: ±5 V) with a 1 Mohm resistor attached to both the positive and negative outputs, and (2) to ensure adequate separation (1.5 or 3 cm) between the dipole pairs, compared to the dipole length (3 mm). Part (1) of the solution ensures a known and matched value for the current in each cable of the dipole pair, and part (2) keeps the current between the pairs one order of magnitude lower than in each dipole.

#### 2.1.5. Synthetic Time-Course Generation

**Figure 2:**
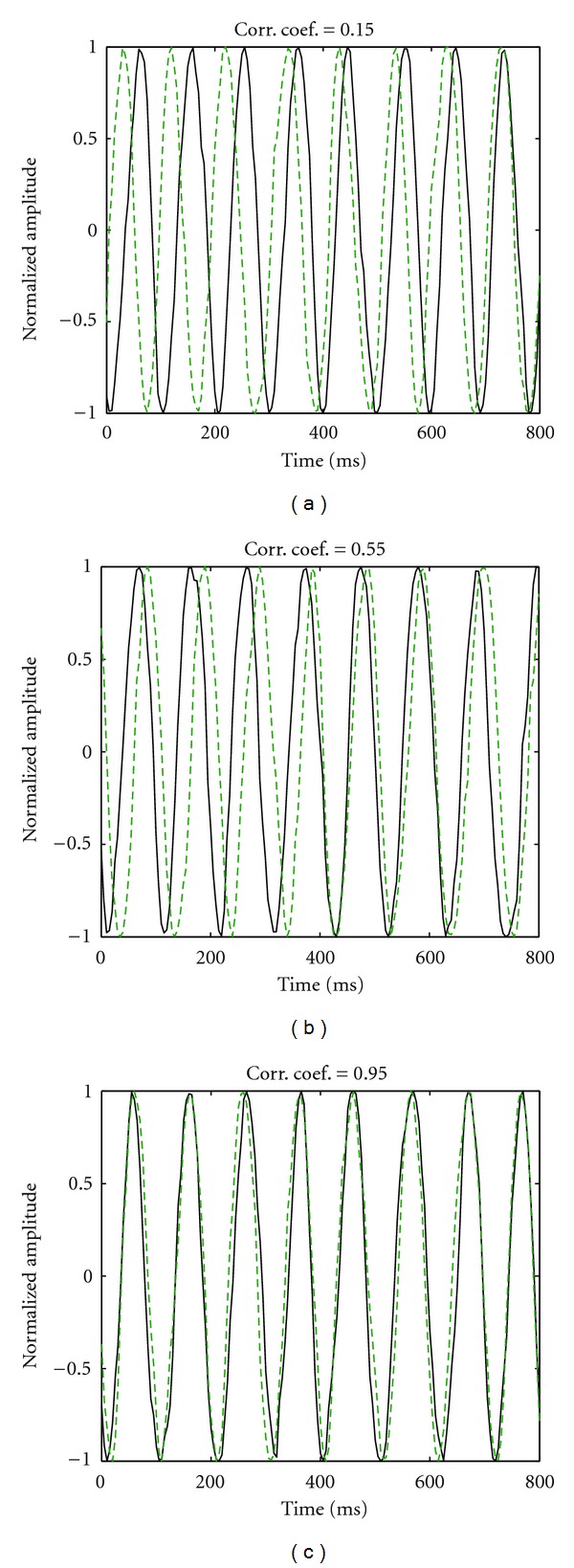
Time-courses for three different levels of correlation. Dashed and solid curves represent the dipole activities on the two source locations. Due to the addition of Gaussian noise, the signals diverge slightly from sine waves. Their phase synchrony grows as correlation increases.

For each time sample, an instantaneous frequency was drawn from a Gaussian distribution centered on a 10 Hz frequency: *N*(10 Hz, 3 Hz). The final time course consisted of the sine of the cumulative sum of such instantaneous frequencies, with a random initial phase. The dipole time courses were controlled either for low (0.15 ± 0.05) medium (0.55 ± 0.05) or high (0.95 ± 0.05) correlation (Figure 2).

**Figure 3:**
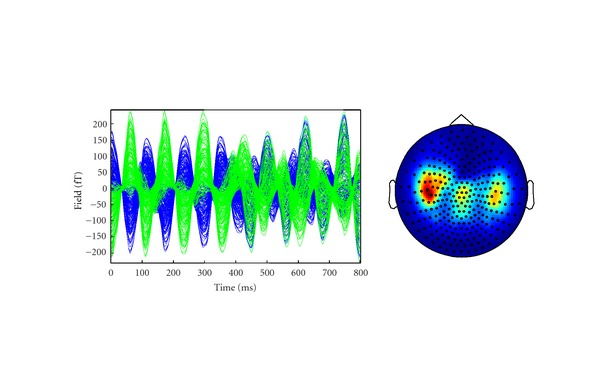
Sensor level plots generated by dipoles in positions #1 and #2, at 15% correlation. (a) signal time courses; (b) topographic plot of signal power at 10 Hz.

In Figure 3 we show a plot of sensor data in the time domain and a sensor-power plot of Fourier transformed data at the frequency of interest of 10 Hz.

### 2.2. Spatial Filters

Since the focus of our work is to quantify the influence of correlation in the localization accuracy of dipoles with known locations, the choice of source mapping strategy was a voxel-wise spatial filter, named beamformer. Parameters for these spatial filters depend both on the forward model chosen (source distribution and a volume conductor model) and the data.

#### 2.2.1. Forward Model

A three-dimensional grid with 5 mm step was employed for source analysis, bounded by a sphere of 65 mm radius centered at the origin. At each grid point (voxel), the full rank-3 leadfield is calculated, and subsequently reduced to rank-2, since in a spherical conductor model the radial component is zero [23]. The volume conductor model is a sphere with a radius of 7 cm.

#### 2.2.2. Linear Constrained Minimum Variance (LCMV) Beamformer

Linear Constrained Minimum Variance beamformers are widely employed both in time and frequency domain [9, 10]. As a first step, the 100 active epochs and the 100 inactive ones are Fourier transformed and then averaged. DICS source power mapping procedure was applied to the data to both averages. Then, the output of the silent average was used as a baseline. The active average was contrasted to the baseline.

LCMV and DICS consist in the following procedure: a filter matrix **A** is employed in a linear transformation from the sensor level to the brain space. **A** filters the source activity (in a given frequency band or time window) at the *i*th voxel (grid point) with unit gain while suppressing contribution from the other voxels. The filter depends on the data by means of the covariance **C**(*t*) (LCMV, time domain) or the cross spectral density (CSD)** C**(*f*) (DICS, frequency domain). Since the two domains are dual, we will define both matrices as **C** in the following description of the filtering procedure. The minimization problem which yields **A** has the following solution:

(1) $$A_{i}={\left(L_{i}^{T}C_{r}L_{i}\right)}^{-1}L_{i}^{T}C_{r}^{-1},$$

where **C**_*r*_ = **C** + *α* **I** and *α* is a regularization parameter. In our case *α* = 15%, the time window of interest *t* was the entire epoch and *f* = 10 ± 3 Hz so that most of the signal information content was considered. **L** is the leadfield matrix. The columns of **L** contain the solution of the forward problem for three orthogonal unit current dipoles placed at the *i*th voxel. However, since the dipole radial component is silent, the leadfield rank at each site is 2. In a spherical conductor, the tangential eigenvectors span the space containing all possible source orientations that can be detected with MEG. The quantity (**L**_*i*_^*T*^ **C**_*r*_ **L**_*i*_)^−1^ is often referred to as the beamformer gain factor.

The source cross-power estimates between the two dipole components at the *i*th voxel are given by:

(2) $$\begin{array}{c} P_{i}\;=\;A_{i}C_{r}A_{i}^{*\text{T}}. \end{array}$$

If the condition *λ*_1_≫ *λ*_2_ holds for the singular values of ***P***, the source can be considered to have a fixed orientation. Otherwise, the power estimate can be obtained by computing the trace of the ***P*** matrix.

In this paper, the implementations of LCMV and DICS present in the Fieldtrip package were employed (http://fieldtrip.fcdonders.nl/).

## 3. Results

### 3.1. Localization Results

**Figure 4:**
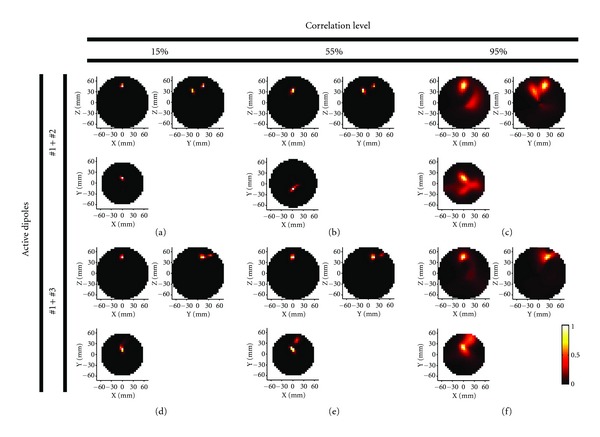
Localization results of the two different dipole couples: couple #1 and #2 (left and right, 3 cm distance, in the higher panel) and couple #1 and #3 (both left, 1,5 cm distance, in the lower panel).

Since the localization results of LCMV and DICS appear extremely similar (as one should expect), we will focus on DICS results. The power mappings of dipoles oscillating with low, medium, and high correlation are shown in Figure 4. In presence of a low correlation level, we obtain a good localization result for both couples of dipoles (absolute maxima on dipole sites, Figure 4(a) (couple #1 + #2) and Figure 4(d) (couple #1 + #3)). Performances decrease only slightly for a correlation level of 0.55 (Figures 4(b) and 4(e)). The localizations are marginally more blurred than in the previous case, and the relative power is faintly reduced. In the case of the close dipoles #1 and #3 one could get the deceptive idea that a 55% source correlation level provides for better results than 15% (Figures 4(d) and 4(e), lower panel, *XY* plane). This is only because source #3 is detected as more blurred, and its presence is perceivable in the lower *XY* plane where the absolute maximum of source #1 is found. In the case of high correlation, the two sources are still recognizable for the couple #1 + #2 (Figure 4(c)) whereas couple #1 + #3 is detected as a single source (Figure 4(f)).

### 3.2. Detected Power Levels at Sites of Interest

**Figure 5:**
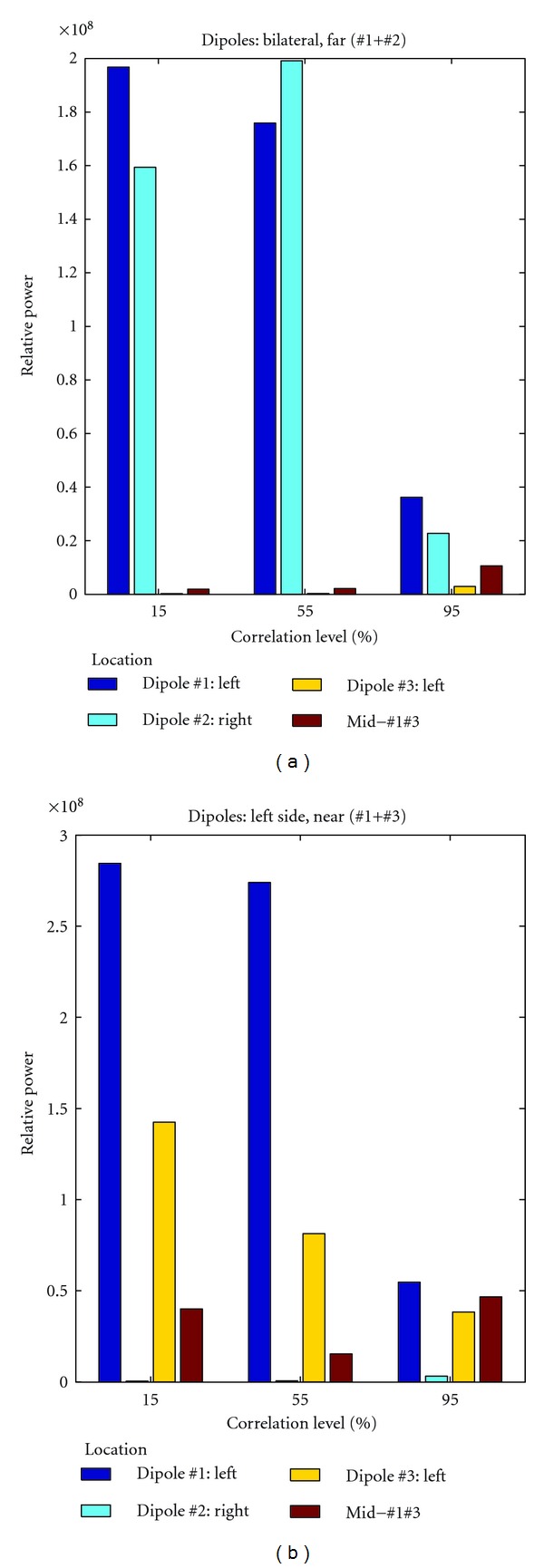
Plots of relative power normalized to the baseline obtained by means of DICS at four sites of interest: location of dipole #1 (deep blue), dipole #2 (light blue), and dipole #3 (yellow) and the median point between dipole #1 and #3 (red). The high relative values are a symptom of the good performance of the spatial filter.

Here we focus on power levels at four locations of interest in order to discern the correlation effects in a realistic environment (Figure 5). The three dipole sites are considered (deep blue, light blue, and yellow). In addition, based on the previous results, we focus on the median point between dipole #1 and #3 (red bar). Due to the spatial proximity, the dipole couple #1 + #3 tends to merge when the correlation degree increases. This does not happen in the case of the couple #1 + #2. In this case the distance always prevents the detection of these dipoles as one merged source. Differently from the couple #1 + #3, the dipoles just attenuate each other's power in the localization process.

For dipoles #1 and #2, the absolute maxima of source power detection are always correct. No relevant contribution to the power mapping is coming from the site between dipoles #1 and #3 as well as from dipole #3 at correlation levels lower than 95%, proving DICS's remarkable spatial accuracy. For high correlation the dipole sites show only 10 to 15% of the power detected in the low correlation simulation. The power level on the other two sites is not irrelevant anymore, if compared to the actual dipole sites.

For dipoles #1 and #3, due to the limited distance, 50% of power of the external source (dipole #3) is not detected at low correlation. At a correlation degree of 55%, the intensity of dipole #3 is 30% of dipole #1, but the low level of power in the site between the dipoles shows that the segmentation between the two detected sources is still acceptable. Only at 95% correlation level, the two sources are not detectable anymore. The power level on the site between #1 and #3 is higher than in site of dipole #3.

Finally, the localization error (LE) for each condition and each source was calculated. Results in Table 1 show that the error is in the order of one voxel up to a correlation level of 55%.

## 4. Discussion

Connectivity studies by means of whole-head non invasive techniques as MEG and EEG are essential to get an insight on brain functional networks. However, the choice of the best algorithm for the network detection is not a trivial task [24]. Among several approaches [25–27], LCMV still stands as one of the most powerful schemes for detection of functional connectivity [11, 28–31]. However, LCMV beamformers are prone to correlation bias effects. Since these effects have been univocally shown only in a PC simulation context, we simulated sources in a human phantom in order to estimate how correlation affects the detection of different network hubs in a real MEG measurement involving every kind of environmental noise. Our findings show that, with data recorded in a real MEG shielded cabin, LCMV suffers from a relevant bias only when the correlation degree between the sources is extremely high. The 55% level is already a remarkably elevated degree of correlation for the detected activity of brain functional networks [32, 33]. It is worth noting that in noninvasive studies the detected time courses of network hub activities in the human brain are reconstructed with some kind of inverse scheme. This implies a certain degree of inaccuracy. Therefore the original correlation of source time-courses could be relatively higher.

It should be noted that this study has different limitations. The number of simultaneously active sources was limited to two in order to study correlation effects in a real recording environment. More complex configurations should be studied in the future. Papadelis and colleagues [22], using a spheroid phantom similar to the one employed in the present study, estimated the localization accuracy of a synthetic-aperture magnetometry (SAM) beamformer [34] when three dipolar sources were simultaneously active. In this case the beamformer failed to detect the third source within 30 mm of the other two. Differently from that study, our results do not show a performance decrease for the deepest source (dipole #2) with respect to the other ones. Our data suggest that MEG should be able to localize 3 cm deep sources under the condition of a sufficient number of trials (i.e., sufficiently high SNR). Furthermore, the use of a spheroid phantom is not optimal for a realistic MEG simulation. A more realistic approach should consider different dipole orientations and time shifts of sources as well as different time-course envelopes. A possible further step in the direction of realistic conditions would be the construction of a human-shaped phantom with different compartments for brain, cerebrospinal fluid (CSF), skull, and scalp. This could potentially yield an increase in the accuracy as shown by the MEG results in [35]. The significance of precise conductivity values for MEG, where only one volume conductor is used, has been downplayed by several studies [35–38]; the shape of the volume conductor plays a more relevant role. The simplest, first-order head model is a sphere; a higher-order model could potentially map more subtleties of the head anatomy. Leahy and colleagues show that with a realistic skull phantom and a corresponding BEM head model, the MEG localization errors are comparable with the potential registration errors. Furthermore, they found out that the localization errors induced by a locally fitted sphere are slightly larger than those generated by a BEM model (3.03 mm *versus* 3.47 mm in the localization error; 6.8°  *versus* 7.7° in the tangential component error). These findings suggest that, while generating a subject-specific volume conductor is probably adding excessive complexity, a standard BEM model scaled to the subject (i.e., a global rescaling transformation with 7 parameters: 6 parameters for rotation and translation and 1 for scaling) could be a fair compromise. This information can be extracted just from the fiducial points of the subject. We performed a brief comparison between a spherical and a “Nolte” model [36]. For the sphere, only rank-2 leadfields were used, for the reasons mentioned in the methods section; this does not necessarily apply to the “Nolte” model. The 7-parameter transformation was then applied to a boundary element method (BEM) model generated from the MRI image of a subject's head, and the ratio between rank-3 and rank-2 leadfields was calculated for each position on a regular grid with 5 mm resolution. The median ratio resulted smaller than 0.1%, indicating that most information input in the model is nondescriptive. Only 3.7% of the sites (435 out of 11654) had a ratio larger than 1%, and less than 0.2% (20 sites) crossed a 5% threshold. This finding suggests that the use of a realistic phantom and a corresponding accurate model appears necessary only in particularly elaborated simulations.

The approach with real current dipoles employed in this study can be further extended in the future to investigate human brain functional networks (resting state, acoustic, sensorimotor, etc.) comparing real and simulated (nonsynthetic) data with the limitations described above. A comparison between human networks and simulated ones at different correlation levels can provide new insights both on the physiological meaning of such human networks and on beamformer limitations as a detection tool for connectivity studies. In fact, the LCMV beamformer can be employed in two simple ways in order to access brain connectivity: 1. In the time domain in order to reconstruct the source time-courses from different areas and in a second step to study their connectivity by means of different algorithms [11, 39–41]. Another possible extension of the present study is the use of DICS not just for power but also for coherence mapping. In this last case, instead of calculating the source power estimates at the *i*th voxel by means of (2), the cross spectrum and coherence estimates between the tangential dipoles at the voxels placed at **r**_1_ and **r**_2_ can be estimated for every brain voxel [10].

## Figures and Tables

**Table 1:** Localization errors (LEs) in the LCMV outputs. Results are acceptable up until a 55% level of correlation. To our surprise, results for source #1 and source #3 (nearby sources) at 55% were slightly better than findings on the same couple of sources with 15% correlation as well as with far sources #1 and #2 simultaneously active at same correlation level (55%). Differences were always in the order of 1 voxel (5 mm). For high correlation between sources (95%), a more relevant error is detectable for nearby sources whereas only a single local maximum is retrievable in the area between the two sources for the far ones.

Correlation level	LE source #1- source #2 (mm)	LE source #1- source #3 (mm)
15%	S1, 5; S2, 5	S1, 5; S3, 10
55%	S1, 11; S2, 9	S1, 5; S3, 7
95%	S1, 15; S2, 12	Single peaks not detectable
